# Effect of *Streptomyces roseolus* Cell-Free Supernatants on the Fungal Development, Transcriptome, and Aflatoxin B1 Production of *Aspergillus flavus*

**DOI:** 10.3390/toxins15070428

**Published:** 2023-06-30

**Authors:** Louise Maud, Florian Boyer, Vanessa Durrieu, Julie Bornot, Yannick Lippi, Claire Naylies, Sophie Lorber, Olivier Puel, Florence Mathieu, Selma P. Snini

**Affiliations:** 1 Laboratoire de Génie Chimique, Université de Toulouse, CNRS, INPT, UPS, 31326 Toulouse, France; louise.maud@toulouse-inp.fr (L.M.); florianboyerpro@gmail.com (F.B.); julie.bornot@toulouse-inp.fr (J.B.); 2 Laboratoire de Chimie Agro-Industrielle (LCA), Université de Toulouse, INRAE, INPT, 4 Allée Emile Monso, 31030 Toulouse, France; vanessa.durrieu@ensiacet.fr; 3 Toxalim (Research Center in Food Toxicology), Université de Toulouse, INRAE, ENVT, EI-Purpan, UPS, 31062 Toulouse, France; yannick.lippi@inrae.fr (Y.L.); claire.naylies@inrae.fr (C.N.); sophie.lorber@inrae.fr (S.L.); olivier.puel@inrae.fr (O.P.)

**Keywords:** *Aspergillus flavus*, *Streptomyces roseolus*, cell-free supernatant, biocontrol, aflatoxin B1, transcriptomic analysis

## Abstract

Crop contamination by aflatoxin B1 (AFB1), an *Aspergillus-flavus-*produced toxin, is frequently observed in tropical and subtropical regions. This phenomenon is emerging in Europe, most likely as a result of climate change. Alternative methods, such as biocontrol agents (BCAs), are currently being developed to reduce the use of chemicals in the prevention of mycotoxin contamination. Actinobacteria are known to produce many bioactive compounds, and some of them can reduce in vitro AFB1 concentration. In this context, the present study aims to analyze the effect of a cell-free supernatant (CFS) from *Streptomyces roseolus* culture on the development of *A. flavus*, as well as on its transcriptome profile using microarray assay and its impact on AFB1 concentration. Results demonstrated that in vitro, the *S. roseolus* CFS reduced the dry weight and conidiation of *A. flavus* from 77% and 43%, respectively, and was therefore associated with a reduction in AFB1 concentration reduction to levels under the limit of quantification. The transcriptomic data analysis revealed that 5198 genes were differentially expressed in response to the CFS exposure and among them 5169 were downregulated including most of the genes involved in biosynthetic gene clusters. The aflatoxins’ gene cluster was the most downregulated. Other gene clusters, such as the aspergillic acid, aspirochlorine, and ustiloxin B gene clusters, were also downregulated and associated with a variation in their concentration, confirmed by LC-HRMS.

## 1. Introduction

Aflatoxin B1 (AFB1) is a mycotoxin produced by several fungal species belonging to *Aspergillus* section *Flavi*, such as *A. flavus*, the most prevalent species. Due to the physiological properties of aflatoxigenic fungal strains, it was admitted a long time ago that AFB1 contamination occurs in tropical and sub-tropical regions [1]. However, due to climate change, the geographical distribution of all mycotoxins is getting increasingly large, leading to an increase in contamination levels or even the appearance of some mycotoxins in areas and in food matrices that were previously considered safe. European countries such as France are therefore facing a serious emerging threat of the appearance of AFB1 in maize, which could cause many health and economic issues [2,3,4].

As AFB1 is the most potent naturally occurring carcinogen causing human hepatocarcinoma; it is classified by the International Agency for Research on Cancer (IARC) in group 1 [5]. Therefore, it is necessary to prevent fungal growth and AFB1 contamination to ensure food safety. Presently, the use of chemical fungicides is favored but may present several disadvantages. Indeed, this chemical method presents not only toxic effects on humans and animals but also harmful effects on the environment, leading to imbalances in microbial ecosystems. Moreover, its effectiveness decreases more and more due to the progressive development of resistance mechanisms in the target organisms that compromise disease control, as is the case of azoles, which are one of the major classes of fungicides used to reduce fungal growth in cereal crops [6,7,8]. This awareness encourages research on and the development of alternative methods to reduce mycotoxin contamination through the use of biocontrol strategies. These strategies can involve macroorganisms, microorganisms, chemical mediators (such as pheromones), and natural compounds of plant, microbial, and mineral origins.

Microorganisms have gained interest due to their ability to produce many metabolites with interesting biological activities. Among microorganisms, the *Streptomyces* genus, belonging to the *Actinobacteria* phylum, has been extensively studied. *Streptomyces* spp. are Gram-positive filamentous bacteria; ubiquitous in soil, they can be endophytic to plants and are found in root systems. There are known to produce a plethora of specialized metabolites, hydrolytic enzymes, and potentially novel anti-microbial compounds [9]. Moreover, some *Streptomyces* strains have been successfully evaluated as biocontrol agents (BCAs) against several phytopathogens. *S. griseoviridis* [10], *S. plicatus* [11], *S. mycarofaciens* [12], and *S. philanthi* [12,13] are some examples of *Streptomyces* strains known to reduce in vitro and/or in vivo contamination by phytopathogenic fungi. Recently, several studies have also demonstrated that volatile organic compounds (VOCs) generated by some *Streptomyces* strains can protect seeds against fungal contamination [14,15,16,17,18]. Despite all these proofs of concept, only two commercial products formulated from alive *Streptomyces* strains are available on the European market (Mycostop^®^ and Actinovate^®^); however, neither of them is specifically registered for AFB1 contamination management. Numerous efforts have been made to select new *Streptomyces* strains that are effective against AFB1 contamination, yet none of the studies led to the commercialization of a specific BCA. On the other hand, some antibiotics, such as dioctatin A, blasticidin A, and aflastatins, produced by *Streptomyces* strains have been demonstrated to inhibit fungal growth and aflatoxin production in *A. parasiticus* [19,20,21,22]. However, these studies are mainly based on phenotypic analyses, and no comprehensive transcriptomics studies have been carried out.

Our previous study demonstrated that the *S. roseolus* strain in co-culture with *A. flavus* can reduce AFB1 concentration with a slight effect on fungal growth [23]. Moreover, the reduction in AFB1 concentration in *A. flavus* grown in co-culture with *S. roseolus* was the result of the downregulation of the expression of genes coding for enzymes involved in AFB1 biosynthesis. The study of the potential of this strain to be a BCA deserves further investigation to understand its complete mode of action. Therefore, the present study aims to investigate the antifungal and anti-aflatoxigenic properties of the cell-free supernatant (CFS) from *S. roseolus* cultures on *A. flavus.* For this, phenotypic, transcriptomic, and metabolomic analyses were performed.

## 2. Results

### 2.1. Effect of CFSs on AFB1 and Aflatoxin B2 (AFB2) Concentrations

**Figure 1:**
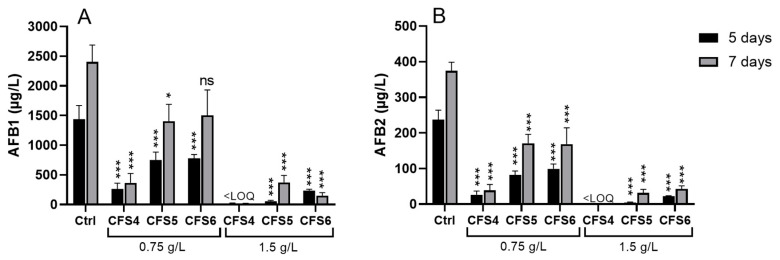
Effect of CFS4, CFS5, and CFS6 at two final concentrations (0.75 and 1.5 g/L) on AFB1 (Panel (**A**)) and AFB2 (Panel (**B**)) concentrations in *A. flavus* cultures incubated 5 days or 7 days at 28 °C on ISP2 medium. Results are expressed as mean ± SEM. One-way ANOVA, Dunett’s multiple comparisons post-hoc test. * *p*-value < 0.05; *** *p*-value < 0.001; ns: not significant; LOQ: limit of quantification.

The addition of each CFS produced in the culture medium resulted in a significant reduction in AFB1 and AFB2 concentrations under almost all tested experimental conditions (Figure 1). It was found that the percentage reduction in AFB1 and AFB2 concentrations was not affected to the same extent by CFS production time (4, 5, or 6 days), CFS concentration (0.75 or 1.5 g/L), and *A. flavus* incubation time (5 or 7 days). Among the three CFSs tested at a concentration of 0.75 g/L, CFS4 led to the best reduction in AFB1 (82 and 85% after 5 and 7 days, compared with their respective control conditions) and AFB2 (89 and 97% after 5 and 7 days) concentrations. In the same way, at a concentration of 1.5 g/L, CFS4 was also the most efficient in decreasing AFB1 and AFB2 concentrations under the limit of quantification (LOQ) after 5 and 7 days of incubation. Based on these results, CFS4 was selected to pursue further investigation.

### 2.2. Effect of CFS4 after Heat Treatments on AFB1 and AFB2 Concentrations

**Figure 2:**
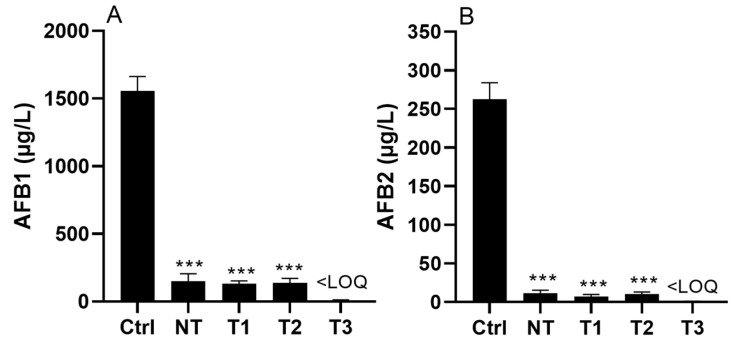
AFB1 (Panel (**A**)) and AFB2 (Panel (**B**)) concentrations in *A. flavus* cultures exposed to CFS4 at 1.5 g/L after heat treatment and incubated for 7 days at 28 °C on ISP2 medium. Ctrl = without CFS4, NT = Not-Treated CFS4, T1 = 100 °C 5 min, T2 = 100 °C 15 min, and T3 = 121 °C 20 min. Results are expressed as mean ± SEM. One-way ANOVA, Dunett’s multiple comparisons post-hoc test. *** *p*-value < 0.001; LOQ: limit of quantification.

Figure 2 presents the results obtained for the effect of CFS4 (1.5 g/L) on the concentrations of AFB1 and AFB2, after heat treatment. *A. flavus* cultures were incubated for 7 days at 28 °C. Three thermic treatments were tested (T1 = 100 °C 5 min, T2 = 100 °C 15 min, and T3 = 121 °C 20 min). It was noted that regardless of the heat treatment applied to CFS4, the latter remained active and allowed a reduction in AFB1 and AFB2 concentrations by more than 90%. When CFS4 was sterilized (T3 = 121 °C 20 min), AFB1 and AFB2 were detected, but their concentrations were under the LOQ.

### 2.3. Effect of CFS4 on Fungal Growth and Sporulation

**Figure 3:**
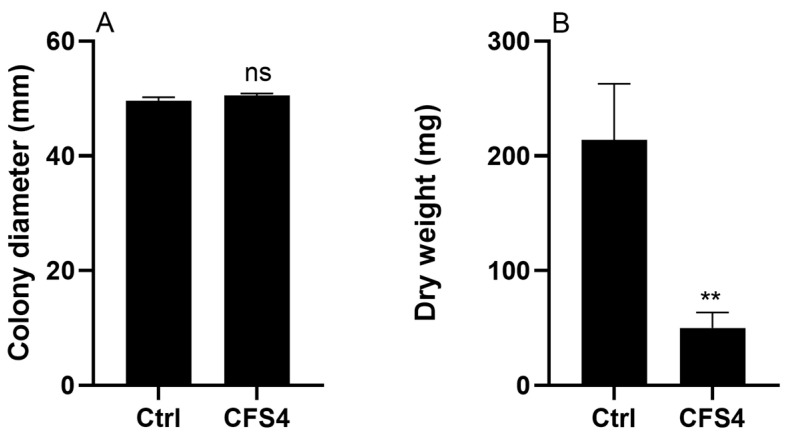
Effect of CFS4 at 1.5 g/L on *A. flavus* growth by measuring the fungal colony diameter in mm (Panel (**A**)) and by measuring the fungal dry weight in mg (Panel (**B**)) after 5 days of incubation at 28 °C on ISP2 medium. Results are expressed as mean ± SEM. Student’s *t*-test. ** *p* < 0.01; ns: not significant.

The effect of CFS4 (1.5 g/L) on *A. flavus* growth after five days of incubation is presented in Figure 3. When fungal growth was evaluated by the measurement of the fungal colony diameter, results demonstrated that the growth was not significantly impacted. However, by using the second method, the measurement of the fungal dry weight, results demonstrated that the fungal growth was significantly reduced (77% of reduction) when *A. flavus* was exposed to CFS4.

**Figure 4:**
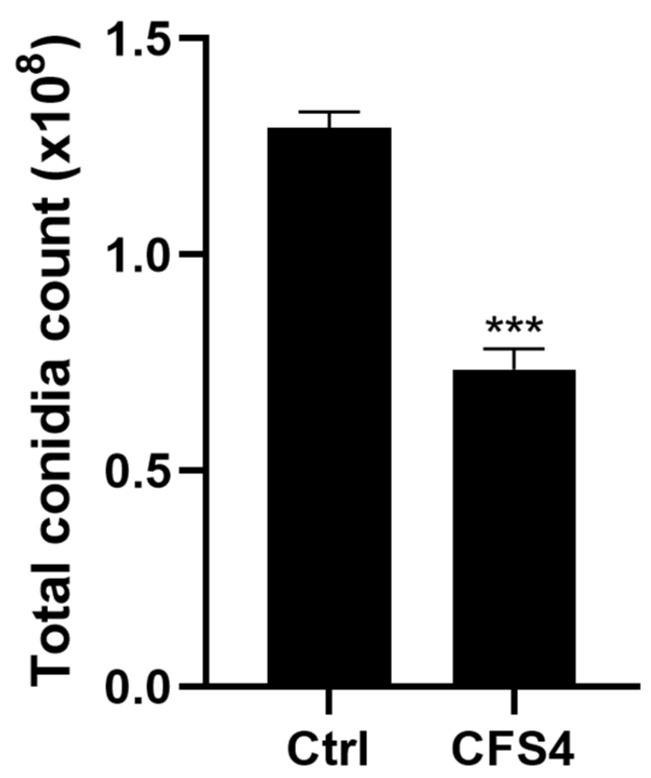
Effect of CFS4 at 1.5 g/L on sporulation of *A. flavus* after 5 days of incubation at 28 °C on ISP2 medium. Results are expressed as mean ± SEM. Student’s *t*-test. *** *p* < 0.001.

The effect of CFS4 (1.5 g/L) on the sporulation of *A. flavus* was investigated by the total conidia count at the end of the incubation time (Figure 4). A significant decrease was observed, with a reduction of 43% (*p* < 0.001) in *A. flavus* culture on medium supplemented with CFS4 at 1.5 g/L.

**Figure 5:**
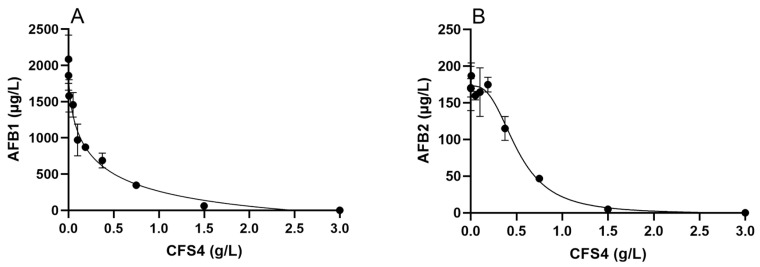
Dose–response curves of the effect of CFS4 on AFB1 (Panel (**A**)) and AFB2 (Panel (**B**)) concentrations in *A. flavus* cultures exposed to CFS4 at several concentrations and incubated for 7 days at 28 °C on ISP2 medium. Results are expressed as mean ± SEM.

To determine the half maximal inhibitory concentration (IC50) of CFS4, several concentrations were tested (0.0005, 0.005, 0.05, 0.1, 0.187, 0.375, 0.75, 1.5, and 3 g/L); the results are presented on Figure 5. Based on these dose–response curves, absolutes of IC50 calculated with the GraphPad Prism 8 software were 0.1418 g/L for AFB1 and 0.5006 g/L for AFB2.

### 2.4. Transcriptomic Response of A. flavus to CFS4 Exposure

**Figure 6:**
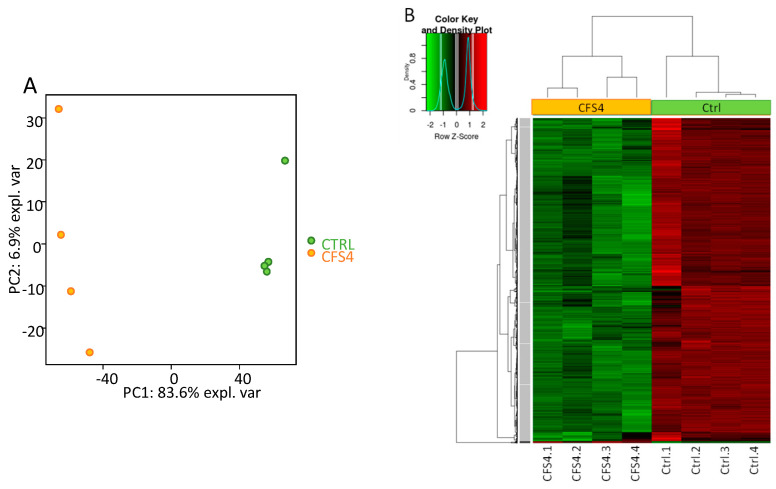
Transcriptomic impact of CFS4 at 1.5 g/L on *A. flavus* cultures incubated for 5 days at 28 °C on ISP2 medium. Microarray data were produced on four replicated *A. flavus* cultures (CTRL and CFS4-treated). Panel (**A**): Projection of samples on principal components 1 (PC1) and 2 (PC2) from a principal component analysis (PCA) of log2FC values. Panel (**B**): Heatmap of the 5198 significantly regulated genes for the CFS4–CTRL comparison. Red and green colors presented in the heatmap indicate values above and below mean-centered and scaled expression values (Z-score), respectively. Black indicates values close to the mean. The gene clustering and individual clustering are illustrated on the left and the top panel dendrogram, respectively. The dark and light gray boxes on the gene dendrogram illustrate the 2 gene clusters showing a specific expression pattern.

To study the impact of CFS4 (1.5 g/L) on *A. flavus* at the transcriptional level, a genome-wide transcriptomic analysis by microarray was performed. A set of 8544 probes (corresponding to 8544 genes) was identified as significantly differentially expressed (FDR < 5%) between the control and the CFS4 exposure condition. Principal component analysis (PCA) performed on the log2FC values of the dataset revealed a clear separation of the control and CFS4 condition, along with the first component, which explained 83.6% of the total variance (Figure 6A). Hierarchical clustering of individual expression values was performed on 5198 significant features for the CFS4–CTRL comparison, based on the FDR method, with a *p*-value and fold change (FC) threshold respectively set to 0.05 and 2 and is presented in Figure 6B. The hierarchical clustering reveals that *A. flavus* exposed to CFS4 displays a distinct expression pattern. Regarding the clustering dendrogram, two gene clusters presenting specific expression profiles were identified. Cluster 1 contains only 29 genes that are upregulated upon CFS4 exposure. Cluster 2 contains 5169 genes that are downregulated upon CFS4 exposure.

#### 2.4.1. Effect of CFS4 on Global Specialized Metabolism

**Figure 7:**
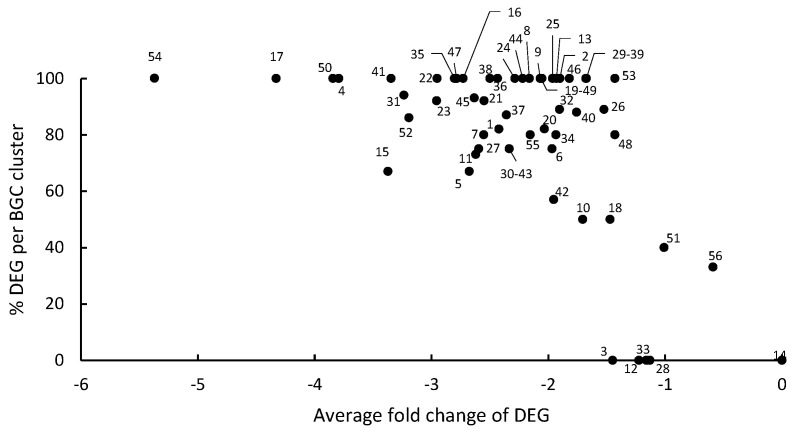
Summarized gene expression regulation of the 56 BGCs of *A. flavus* exposed to CFS4 (numbered from 1 to 56). The *x*-axis represents the average fold change in each BGC and the *y*-axis represents the percentage of differentially expressed genes (DEGs) within all genes of the BGC (number of DEG/number of detected genes).

The impact of CFS4 on the expression of genes belonging to 56 described or predicted BGCs was analyzed. Figure 7 summarizes gene expression regulation of the 56 BGCs of *A. flavus* exposed to CFS4. A total of 54 were downregulated, and 24 of them had 100% of their genes significantly impacted by CFS4. Cluster 54, encoding genes involved in aflatoxin biosynthesis, was the most downregulated BGC and exhibited fold changes (FC) up to −12 with an average fold change of −5.37.

To date, 15 BGCs have been associated with the production of specific specialized metabolites. According to the numbering in Georgianna et al.’s work [24], the clusters are as follows: 4 (ditrytophenaline—[25]), 11 (aspergillic acid—[26]), 15 and 32 (aflatrem—[27]), 21 (aspirochlorine—[28]), 23 (leporins—[29]), 27 (asparasone—[30]), 31 (ustiloxin B—[31]), 35 and 48 (piperazines—[32]), 39 (aflavarin—[33]), 54 (aflatoxins—[34]), 55 (cyclopiazonic acid—[35]) and 56 (kojic acid—[36]). The 15th cluster concerned is the imizoquin cluster [37], which is not numbered in Georgianna et al. but is numbered 44 in Wu et al. [38]. In our study, the data analysis was focused primarily on the expression of genes belonging to these 15 BGCs (Supplementary Materials Table S1); moreover, the production of some corresponding specialized metabolites has also been evaluated by liquid-chromatography high-resolution mass spectrometry (LC-HRMS) (Supplementary Materials Table S2).

#### 2.4.2. Effect of CFS4 on Characterized Specialized Metabolites

➢ Aflatoxins

**Figure 8:**
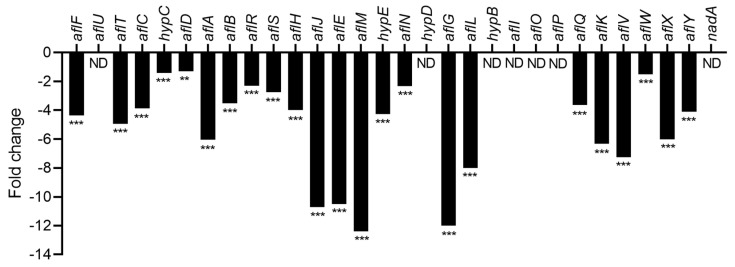
Fold change expression of aflatoxin gene cluster in response to CFS4 exposure. *** *q*-value < 0.001; ** *q*-value < 0.01; ND: not detected.

Aflatoxin biosynthesis from acetyl-CoA involves 27 enzymes encoded by genes gathered in a cluster of 30 genes. The three remaining genes code for a specific transcription factor (AflR), its co-activator (AflS), and a MFS transporter (AflT). The transcriptomic analysis revealed that after CFS4 exposure, all cluster genes were significantly downregulated (Figure 8). The reduction ranged from 1.29 (*aflD*) to 12.38 (*aflM*) fold and genes involved in the intermediate steps of the aflatoxins’ biosynthetic pathway were the most downregulated (from 6.05 for *aflA* to 12.38 for *aflM*). The *aflG* gene, coding for the enzyme responsible for averantin conversion showed a FC decrease of 11.99. Moreover, the *aflM* gene encoded a ketoreductase, an enzyme responsible for the conversion of versicolorin A in dimethylsterigmatocystin and versicolorin B in dihydromethylsterigmatocystin, and which had a FC decrease of 12.38 [39]. LC-HRMS analysis revealed that the downregulation of the aflatoxins’ gene cluster was linked to a 95% decrease in AFB1 relative abundance when *A. flavus* was exposed to CFS4, in comparison with the control culture (Supplementary Materials Table S2).

➢ Cyclopiazonic acid

**Figure 9:**
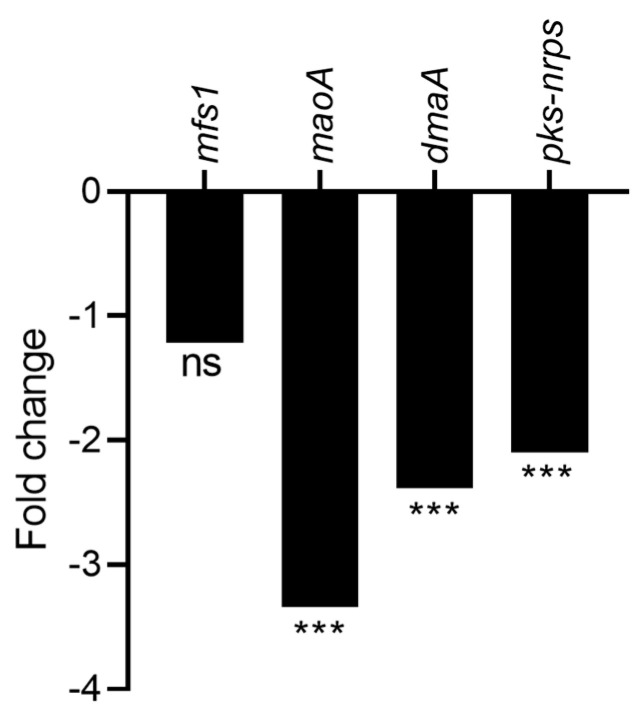
Fold change expression of cyclopiazonic acid gene cluster in response to CFS4 exposure. *** *q*-value < 0.001, ns: not significant.

Cyclopiazonic acid (α-cyclopiazonic acid, CPA) is an indole-tetramic acid mycotoxin whose genes are involved in its biosynthesis and are located directly adjacent to aflatoxins’ biosynthetic pathway cluster in the sub-telomeric region of chromosome 3 [35]. Among the four genes involved in CPA biosynthesis, three of them were significantly downregulated with a fold change from −3.3 for monoamine oxidase (*maoA*), responsible for the conversion of β-CPA to α-CPA in the last step of the biosynthesis pathway, to −2.1 for the hybrid backbone enzyme (PKS-NRPS) involved in the formation of the first stable intermediate in CPA biosynthesis, cyclo-acetoacetyl-L-tryptophan (cAATrp) (Figure 9). However, the LC-HRMS analysis did not reveal any significant difference in the relative abundance of CPA between both experimental conditions (Supplementary Materials Table S2), which may be explained by other regulation mechanisms currently unknown.

➢ Aspergillic acid

**Figure 10:**
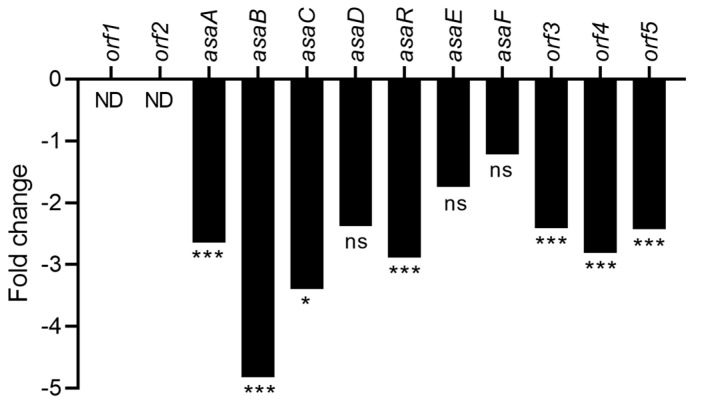
Fold change expression of the aspergillic acid gene cluster in response to CFS4 exposure. * *q*-value < 0.05, *** *q*-value < 0.001. ns: not significant; ND: not detected.

Aspergillic acid is a toxic hydroxamic-acid-containing pyrazinone derived from L-leucine and L-isoleucine residues [40]. Twelve genes gathered in a cluster are involved in aspergillic acid biosynthesis [26]. Transcriptomic analysis revealed that the expression of seven of those genes was significantly downregulated (fold change from −4.8 to −2.3) (Figure 10). For instance, *asaR,* which encodes the specific transcription factor that regulates aspergillic acid production but was found to be unessential once deleted, resulted only in a reduction in ferriaspergillin levels. On the other hand, *asaA,* which is responsible for encoding a putative ankyrin domain protein, was found to be essential since, if deleted, it inhibited the production of ferriaspergillin. The observed fold changes were found to be −2.9 and −2.6, respectively, which might also explain the downregulation of all the genes of the cluster [26]. Interestingly, the downregulation of many genes in the aspergillic acid cluster was not linked to a decrease in the aspergillic acid concentration in *A. flavus* culture exposed to CFS4. Indeed, LC-HRMS analysis revealed that the relative abundance of aspergillic acid was nine-fold higher for *A. flavus* exposed to CFS4 than under the control condition (Supplementary Materials Table S2). This might be explained by the downregulation of the *asaB* gene, coding for a hydroxylase, and responsible for the conversion of aspergillic acid to hydroxyaspergillic acid. However, the relative abundance of hydroxyaspergillic acid was not examined during the LC-HRMS analysis.

➢ Aspirochlorine

**Figure 11:**
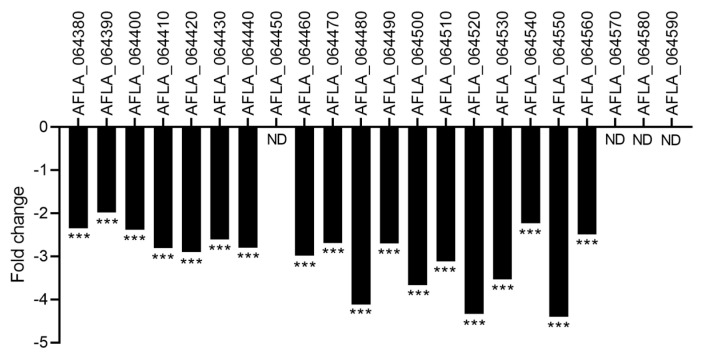
Fold change expression of the aspirochlorine gene cluster in response to CFS4 exposure. *** *q*-value < 0.001. ns: not significant; ND: not detected.

Aspirochlorine is an epidithiodiketopiperazine (ETP) toxin mainly produced by *A. oryzae* and also by *A. flavus*. By combining genetic and chemical analyses, the cluster of genes involved in aspirochlorine biosynthesis was identified in *A. oryzae*. The putative aspirochlorine biosynthetic gene cluster in *A. flavus* was identified based on NCBI gene annotation and ACT/BLASTx comparison and gathered 22 genes from AFLA_0643380 to the AFLA_064590 locus [28]. Transcriptomic analysis revealed that after CFS4 exposure, all genes in the cluster were significantly downregulated (fold change from −4.4 to −1.9) (Figure 11). LC-HRMS analysis revealed that the relative abundance of aspirochlorine was 1.5-fold lower in *A. flavus* culture exposed to CFS4 in comparison with the control condition (Supplementary Materials Table S2).

➢ Ustiloxin B

**Figure 12:**
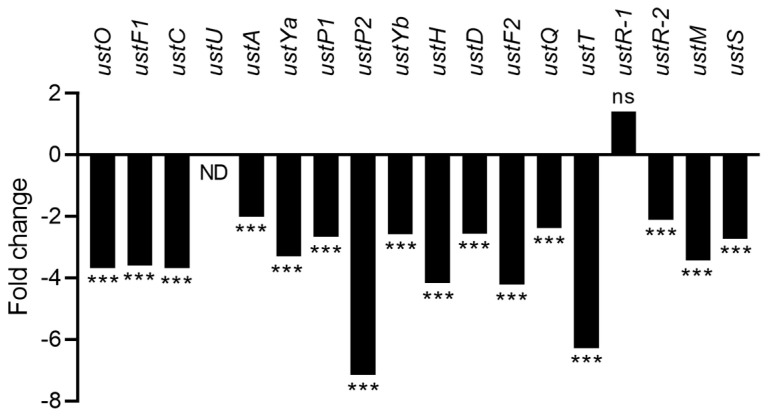
Fold change expression of the ustiloxin B gene cluster in response to CFS4 exposure. *** *q*-value < 0.001. ns: not significant; ND: not detected.

Ustiloxin B is a cyclic tetrapeptide of Tyr-Ala-Ile-Gly. The tyrosine is modified with a methyl group, a hydroxyl group, and a non-protein-coding amino acid, norvaline. Ustiloxin B is synthesized by a ribosomal peptide synthetic (RiPS) pathway, and its biosynthetic gene cluster gathered 18 genes designated as *ustO* (AFLA_094940) to *ustS* (AFLA_095110) [31,41,42]. In this cluster, the exposure to CFS4 led to a significant downregulation of all genes (fold change ranged from −7.1 (*ustP2*) to −2 (*ustA*)) (Figure 12). The *ustR-1* gene (AFLA_095080) was the only upregulated gene in this cluster; it was not significant and *ustU* was not detected. The *ustR-1* gene (AFLA_095080) and *ustR-2* (AFLA_095090), which is downregulated (fold change −2.1), encode for the specific regulator of the cluster, a Zn(II)2Cys6 transcription factor. Indeed, *ustR-1* (AFLA_095080) and *ustR-2* (AFLA_095090) were concatenated to a single gene [41]. Thus, the downregulation of *ustR-2* can explain the downregulation of the whole gene cluster. Ustiloxin B was not detected by LC-HRMS analysis under CFS4 and control conditions.

#### 2.4.3. Effect of CFS4 on Gene Expression Related to Fungal Development

**Figure 13:**
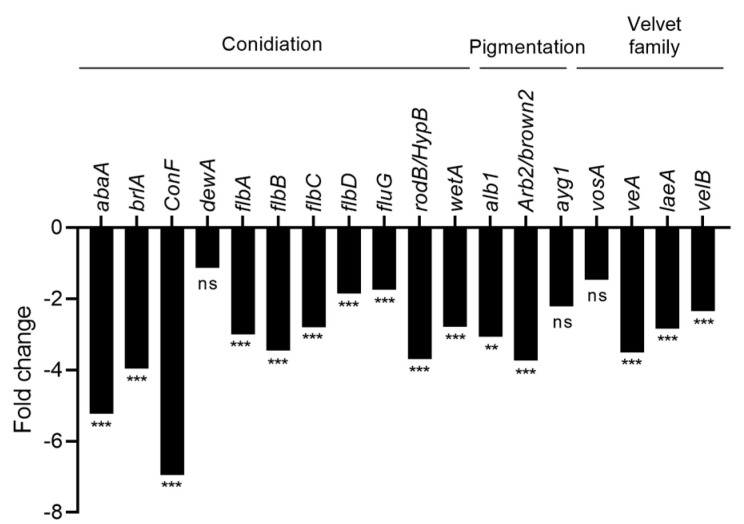
Fold change expression of genes involved in fungal development in response to CFS4 exposure. ** *q*-value < 0.01. *** *q*-value < 0.001. ns: not significant.

As CFS4 modulates fungal development (dry biomass and conidiation), the expression of genes involved in this function was analyzed. The expression level of several genes implicated in conidiation, pigmentation, and of the velvet family was significantly downregulated when *A. flavus* was exposed to CFS4 (Figure 13). Among them, *brlA*, a key transcription factor for the initiation of conidiophore formation [43,44], has a fold change of −3.96. Similarly, *abaA*, implicated in the phialid and metula formation [45], and *wetA*, in spore maturation [38], were both downregulated, with a fold change of −5.29 and −2.79, respectively. Concerning pigmentation, *Arbr2/brown2* was the most downregulated gene, with a fold change of −3.73. Finally, *veA*, *laeA*, *velB,* and *vosA,* belonging to the velvet family, were also downregulated, with fold changes from −2.35 to −3.51.

#### 2.4.4. Effect of CFS4 on Gene Expression of Genes Involved in Fungal Response to External Stimuli

**Figure 14:**
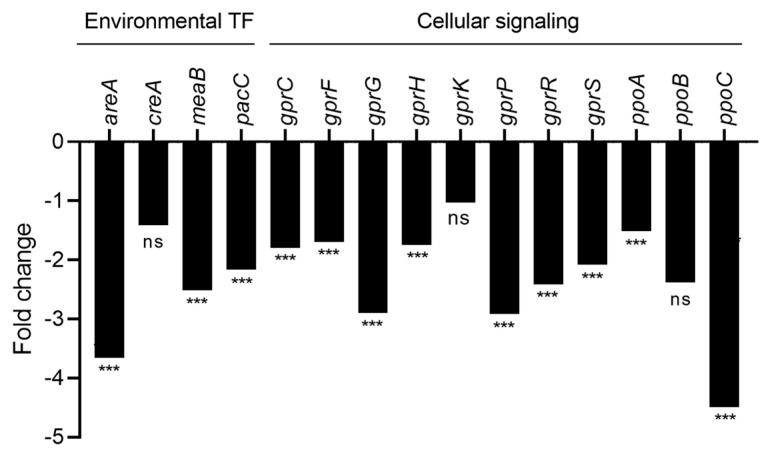
Fold change expression of genes involved in environmental responses and cellular signalization. *** *q*-value < 0.001. ns means “no significant”.

In response to CFS4 exposure, many genes involved in cellular signaling and coding for environmental transcription factors (TFs) were downregulated. Most notable are the *pacC* gene, implicated in the response to the pH environment, with a fold change of −2.16 (Figure 14), and *areA*, a nitrogen regulatory gene, with a fold change of −3.66. Furthermore, a similar effect was observed for *ppoA*, *ppoB*, and *ppoC,* encoding three fatty acid oxygenases, involved in asexual development [46]. A downregulation of *ppoA* and *ppoC* with a fold change of −1.51 and −4.49, respectively, was observed. Regarding the G-protein-coupled receptor (GPCR) genes, coding for GPCR transmembrane receptors, they were all downregulated with a fold change from −1.70 to −2.92, except for *gprK*.

## 3. Discussion

In this study, the effect of *S. roseolus* CFS on fungal development, transcriptome, and AFB1 production of *A. flavus* was studied in an attempt to decipher the mechanism of action of this promising source of bioactive molecules to control AFB1 contamination.

First, the three *S. roseolus* CFSs produced (4, 5, and 6 incubation days) and both CFS concentrations tested (0.75 and 1.5 g/L) were able to reduce AFB1 and AFB2 concentrations in *A. flavus* culture (Figure 1). Nevertheless, the CFS produced after 4 days of incubation at 28 °C (CFS4) was the most efficient in reducing AFB1 concentration, reaching concentrations below the limit of quantification and linked to a downregulation of the aflatoxins’ gene cluster. On the one hand, this suggests that the proportion of bioactive molecules in a CFS is higher after 4 days of incubation in agitated liquid culture than after 5 or 6 days of incubation. On the other hand, it might also indicate that CFS composition differs based on the culture conditions. A difference in the nature of molecules produced by *Streptomyces* sp. HU2014 was observed by spectrophotometric analyses of cell-free filtrate (CFF) after different incubation times [47]. Indeed, it has been described that *Streptomyce*s strains arborized a complex life cycle, which is closely related to specialized metabolite production [48]. The bioactive molecules produced depend on both culture conditions and strains [47,49,50,51]. Moreover, according to Campos-Avelar et al. (2021), among the 59 *Streptomyces* isolates tested, all of them can reduce AFB1 concentration in co-culture with *A. flavus*. However, only the cell-free extracts (CFE) from 31 isolates, produced at 5 days at 25 °C and tested at 10% in the culture medium, were able to reduce AFB1 concentration to a lesser extent [52]. Another study revealed that *S. philanthi* RL-1-178 CFS, produced for 10 days at 30 °C, reduced AFB1 concentration in *A. flavus* and *A. parasiticus* cultures by 96.7 and 86.7%, respectively [53].

Furthermore, a dose effect of CFS4 was demonstrated (Figure 5). The IC50 values of CFS4 were 0.14 g/L and 0.50 g/L for AFB1 and AFB2, respectively, suggesting that CFS4 was less efficient at reducing AFB2 concentration. In the same way, the CFE of *Streptomyces* isolates tested by Campos-Avelar et al. (2021) was also less efficient in reducing AFB2 concentration, although *Streptomyces* isolates had a stronger effect on AFB2 concentration than on AFB1 in co-culture with *A. flavus* [52]. To compare, the aqueous extract of *Mimosa tenuiflora* and *Minthostachys mollis,* both aqueous plant extracts, reduced AFB1 concentration from *A. flavus* with an IC50 of 0.15 g/L and 0.4 g/L, respectively, which are values very close to those found in this study [54,55]. All these data demonstrate the efficiency of *S. roseolus* CFS in reducing AFB1 and AFB2 concentrations in vitro. Finally, heat treatments had no impact on CFS4 efficiency, indicating that the active compounds were not of protein nature but were rather thermostable chemical molecules. In the same way, the fungicide crude extract from the *Pseudomonas aeruginosa* K-187 broth culture conserved more than 90% of its activity against *Fusarium oxysporum*, *A. fumigatus*, and *A. parasiticus* after 100 °C for 30 min [56].

*S. roseolus* CFS4 reduced the dry weight of *A. flavus* by 77% compared with the control without any effect on the colony diameter (Figure 3), revealing the importance of using the dry biomass as an indicator of fungal growth rather than measuring the colony’s diameter. Indeed, many characteristics, such as the length and diameter of hyphae and hyphal density, do not impact colony diameter but can impact dry biomass [57]. Many studies revealed the impact of different actinobacterial strains on *A. flavus* growth and more generally on several phytopathogenic fungi. Indeed, Rahila et al. demonstrated that *Streptomyces chrestomyceticus* STR-2 was able to reduce *Magnaporthe oryzae* growth with a growth inhibition of 50% [58]. Moreover, by using the same method, Shahid et al. (2021) revealed that five actinobacterial strains (*Amycolatopsis pretoriensis* MSCA21, *Saccharopolyspora shandongensis* MSCA89, *Kribbella karoonensis* MSCA185, *S. amritsarensis* V31, and *S. amritsarensis* V4) reduced *Rhizoctonia solani*, *Alternaria alternata*, *A. flavus*, *Fusarium oxysporum f.* sp. *Lycopersici*, *Sarocladium oryzae*, and *Sclerotinia sclerotiorum* growth with a percentage of inhibition from 44.8 to 90% [59]. Natural products are also known to have antifungal activities. An inhibition of 77.1% of *A. flavus* colony diameter was observed when the fungus was treated with honokiol, a phenolic compound in *Magnolia officinalis,* at 100 µg/mL for 72 h at 28 °C, or when it was treated with piperine, a component of long black pepper, at 0.17 mM, with a growth inhibition of 35% [60,61].

In the present study, another morphological change was the decrease in conidiation when *A. flavus* was exposed to CFS4 (Figure 4). It was also evaluated by Zhang et al. with honokiol at concentrations ranging from 50 to 200 µg/mL [61]. This phenotype was correlated with the effect of CFS4 on many genes involved in asexual development. The global transcriptomic analysis revealed a down-expression of *brlA, wetA*, and *abaA* (Figure 13), three transcription factors involved in conidiophore production, the asexual reproductive organs in *Aspergilli* species [44].

Following the same trend, *ppoA, ppoB*, and *ppoC,* which code for fatty acid oxygenases, were also impacted by the CFS4 treatment (Figure 14). A study reveals that a *∆ppoA∆ppoB∆ppoC* triple mutant favors the exacerbated sexual reproduction in *A. nidulans* [62]. Moreover, this triple mutation leads to an inability of *A. nidulans* to produce sterigmatocystin, thus demonstrating the implication of *ppoA, ppoB*, and *ppoC* in specialized metabolism [63]. Furthermore, *conF*, the most downregulated developmental gene, is expressed in conidia and is involved in conidia germination, and its deletion is responsible for the high resistance of conidia to desiccation [64]. The decrease in conidiation observed in this study is very interesting because it limits the dissemination capacity of the fungus and thus limits crop contamination. Interestingly, in a previous study, the co-culture of *S. roseolus* and *A. flavus* induced a hypersporulation of the fungus by up to 36% compared with the control condition (*A. flavus* alone). This phenotype was linked to the overexpression of *brlA*, *abaA*, *fluG,* and *flbA* genes involved in the conidiation process, and more specifically, in conidiation initiation and conidiophore development in *A. nidulans* [23,65,66]. This reveals that the indirect or direct interaction between *S. roseolus* and *A. flavus* leads to a different response of *A. flavus*, highlighting probably a different mechanism of action.

The global transcriptomic analysis conducted in the present study demonstrated that the reduction in AFB1 concentration in *A. flavus* is consistent with a drastic downregulation of the entire aflatoxins’ gene cluster (Figure 8) as already demonstrated in several studies [23,54,60,67,68,69]. Indeed, genetic regulation of aflatoxin biosynthesis is widely described, involving *aflR* and *aflS*. These genes are responsible for coding the specific transcription factor and transcription enhancer, and for regulating the expression of other genes in the aflatoxins’ gene cluster with the exception of *aflT.* This gene is known to encode a fungal transporter that belongs to the major facilitator superfamily (MFS) [39,70]. However, in the present study, *aflT* was severely repressed, which suggests another regulation mechanism, also observed when *A. flavus* was treated with an aqueous extract of *Micromeria graeca* at 10 mg/mL [71]. Caceres et al. (2018) demonstrated that genes involved in the intermediate and late steps of the aflatoxins’ biosynthesis pathway were the most downregulated, even though the entire cluster was impacted. In our study, all genes of the aflatoxins cluster were impacted, with genes encoding enzymes involved in the intermediate stages of the biosynthesis pathway being the most impacted, such as *aflJ* and *aflM*. This downregulation led to a decrease in AFB1 concentration in *A. flavus* culture. Different steps of the aflatoxin biosynthesis pathway can be impacted, leading to a decrease in AFB1 production. Indeed, a downregulation of the genes involved in the late steps of the aflatoxins’ biosynthesis pathway was observed when *A. flavus* was grown in the presence of *Lactobacillus plantarum* [72]. Conversely, genes involved in the early and middle stages of the aflatoxins’ biosynthesis pathway were more downregulated by *A. oryzae* filtrate treatment on *A. flavus* culture [73]. Another study revealed that *S. yanglinensis* in co-culture and its culture filtrate (CF) were able to reduce *A. flavus* growth and AFB1 concentration compared with the control condition by downregulating *aflR* and *aflS* expression, both specific transcription factors of aflatoxins’ biosynthesis [74]. Last, it will be important to check whether a more toxic intermediate has not been accumulated due to the interruption of the biosynthetic pathway. Indeed, the downregulation of *aflM*, encoding a ketoreductase involved in the conversion of versicolorin A (VERA) into demethylsterigmatocystin (DMST), can cause an accumulation of VERA, which is more cytotoxic than AFB1 [75].

The specialized metabolism of *A. flavus* can be modulated by numerous transcription factors sensitive to environmental stimuli, such as CreA, AreA, and PacC. In this study, *S. roseolus* CFS4 has strongly impacted *A. flavus* transcriptome, notably by destabilizing its nutrition process. Indeed, the *creA* gene, involved in the catabolite carbon repression but also in the regulation mechanism of aflatoxin biosynthesis, was downregulated. The inhibition of the aflatoxins’ gene cluster was also observed in a *∆creA* mutant [76,77]. An opposite effect was observed when *A. flavus* was co-cultivated with *S. roseolus* [23]. Thus, it seems that the downregulation of *creA* is not responsible for the downregulation of aflatoxins’ gene cluster. Moreover, *areA*, a nitrogen-regulatory gene, was also downregulated by CFS4. Finally, all *gpr* genes, coding for GPCR transmembrane receptors, were negatively impacted by CFS4 (Figure 14). GPCRs are closely related to the response to external stimuli, and their downregulation can impact the entire primary metabolism of fungi [78]. Furthermore, it has been demonstrated that *grpA* and *gprK* play a role in the repression of aflatoxin production [79].

The *veA* gene, a global regulator of the velvet family, composed of *vosA*, *laeA*, and *velB*, is also downregulated by CFS4 treatment (Figure 13). This gene is known to modulate the specialized metabolism of *Aspergillus* species and to contribute to the virulence of *A. flavus* in vitro and *in planta*. Moreover, aflatoxin production and conidiation were altered in a *∆vea* mutant inoculated on peanuts, corn, and cotton seeds [80]. It has also been demonstrated that the deletion of *veA* leads to a decrease in conidiophore formation in *A. niger* and to a decrease in the conidia number [81]. Furthermore, *veaA* is required for CPA, aflatrem, and aflatoxin production in *A. flavus* [82]. A downregulation of *veA* was also observed when *A. flavus* was treated with piperine or grown with *Bacillus velenzis* E2 [60,83]. However, an overexpression of *veA* was observed during a co-culture with *S. roseolus* and *A. flavus*, or when *A. flavus* was grown in the presence of an aqueous extract of hyssop (*Micromeria graeca*) linked to a downregulation of aflatoxins’ gene cluster [23,71]. Our study also demonstrated a downregulation of the CPA cluster (Figure 9), involved in the biosynthesis of an indole-tetramic acid mycotoxin, and notably the *dmaA* gene. This gene, also called *dmaT*, was upregulated when *A. flavus* was cultivated with *S. roseolus* [23]. The downregulation of the CPA cluster was not correlated with a diminution of CPA-relative abundance, suggesting that the downregulation was not sufficient to ensure a reduction in CPA concentration. In the same way, among the twelve genes involved in the aspergillic acid cluster, seven were significantly downregulated (Figure 10), notably *asaB*, coding for a GA4 desaturase family protein. This gene was also downregulated when *A. flavus* was grown with dimethyl sulfoxide (DMSO) at a concentration of 281.6 mM [69]. Nevertheless, the LC-HRMS analysis revealed that the relative abundance of aspergillic acid was nine-fold higher in *A. flavus* exposed to CFS4 than under control conditions, suggesting a more complex mechanism of regulation. However, this increase in aspergillic acid relative abundance must be taken into consideration because of its potential toxicity. Indeed, previously studied for its antibiotic properties, aspergillic acid exhibits acute toxicity to mice by damaging the nervous system [84].

## 4. Conclusions

In the present study, the impact of *S. roseolus* CFS on *A. flavus* and AFB1 concentration was analyzed. To determine the mechanism of action, morphological and transcriptional analyses of *A. flavus* in the presence of CFS were performed. These experimentations revealed that the decrease in AFB1 concentration was linked to a downregulation of the aflatoxins’ gene cluster. Furthermore, the down expression of genes involved in the conidiation process such as *brlA, wetA* and *abaA* led to a decrease in conidiation. Morphological differences observed on *A. flavus* in co-culture with *S. roseolus* or exposed to its CFS suggest a difference in the mechanism of action, even if the effect on the aflatoxins’ gene cluster was the same. The reduction in conidiation and aflatoxin production is encouraging, and it allows us to consider *S. roseolus* CFSs as a source of bioactive molecules to reduce AFB1 contamination, notably in crops or during grain storage. To achieve this, it is necessary to scale up the process of CFS production, notably in mechanically agitated bioreactors. Formulation assays are in progress, and in vivo and *in planta* tests, notably on maize grain and on the maize plant, are also considered.

## 5. Materials and Methods

### 5.1. Chemical Reagents

AFB1 and aflatoxin mix (AFB1 (1 µg/mL), AFB2 (3 µg/mL), AFG1 (1 µg/mL), and AFG2 (3 µg/mL)) solutions were purchased from Merck KGaA (Saint-Quentin-Fallavier, France). High-performance liquid chromatography (HPLC) and solvents of analytical-grade quality were purchased from Thermo-Fisher Scientific (Illkirch, France). Ultrapure water used for HPLC and molecular biology procedures was purified at 0.22 µm by an ELGA purification system (ELGA LabWater, High Wycombe, UK).

### 5.2. Microbial Strains

The *Streptomyces roseolus* strain was previously identified and selected for its capacity to reduce AFB1 and AFB2 concentrations in *A. flavus* cultures [23,67,85]. The *Aspergillus flavus* NRRL 62477 strain, producer of AFB1 and AFB2, was isolated from paprika samples harvested from a Moroccan market [86]. Both microbial strains were maintained on a solid ISP2 medium (4 g/L α-D glucose; 10 g/L malt extract; 4 g/L yeast extract; 20 g/L agar; pH 7).

### 5.3. S. roseolus Cell-Free Supernatants (CFSs) Production

First, *Streptomyces roseolus* was cultured on a solid ISP2 medium at 28 °C until sporulation (10–12 days). Then, to obtain a liquid pre-culture, a 250 mL Erlenmeyer flask filled with 50 mL of ISP2 medium was inoculated with *S. roseolus* spores recovered from the previous solid culture and incubated at 28 °C in an orbital incubator set at 200 rpm for 48 h. At the end of the incubation time, a 500 mL Erlenmeyer flask filled with 100 mL of liquid ISP2 medium was inoculated with 5 mL of the liquid pre-culture and incubated at 28 °C in an orbital incubator set at 200 rpm. The incubation times were different (4 days (CFS4), 5 days (CFS5), or 6 days (CFS6)) according to the conducted experiment. At the end of the incubation time, liquid cultures were centrifuged at 10,000× *g* for 10 min; then, supernatants were filtered through a 0.22 µm cellulose acetate filter to eliminate bacterial cells. All produced CFSs were stored at −20 °C until use.

### 5.4. Determination of CFSs Dry Matter Content

The dry matter content of each CFS produced was determined after each production trial. Briefly, 3 mL of CFS was weighed before and after drying in an oven set at 103 °C for 24 h. Each measurement was made in triplicate. The difference between before and after drying refers to the CFS dry matter content expressed in g/L.

### 5.5. Effect of CFSs on AFB1 and AFB2 Concentrations

To evaluate the effect of CFS on AFB1 and AFB2 concentrations, *A. flavus* was inoculated in Petri dishes filled with solid ISP2 medium supplemented with CFSs (final volume 20 mL). The volume of CFS added in the ISP2 medium was determined on the basis of their dry matter content to obtain the desired concentrations. Two CFS concentrations were used in the study: 0.75 and 1.5 g/L and several CFSs produced at different times (CFS4, CFS5, and CFS6) were tested. Petri dishes were centrally inoculated with 10 µL of *A. flavus* spore suspension calibrated at 10^6^ spores/mL and incubated for 5 or 7 days at 28 °C.

The effect of CFS4 on AFB1 and AFB2 concentrations was also evaluated after heat treatments. Three heat treatments were tested: 100 °C for 5 or 15 min and 121 °C for 20 min. After heat treatment, CFS4 was added to the ISP2 medium at the final concentration of 1.5 g/L before medium solidification. Then, *A. flavus* was centrally inoculated, as previously described, and incubated for 7 days at 28 °C. At the end of the incubation, AFB1 and AFB2 were extracted, as described below. As control conditions, *A. flavus* was inoculated on a not-supplemented solid ISP2 medium (Ctrl). All experiments were conducted twice in triplicate.

### 5.6. Effect of CFS4 on Fungal Growth and Sporulation

#### 5.6.1. Effect on *A. flavus* Growth

To evaluate the effect of CFS4 on *A. flavus* growth, two methods were followed. The first one was by measuring the fungal colony diameter (mm) at the end of the incubation. The second consisted of measuring the dry weight (g/L) of the fungal culture at the end of incubation. For that, a cellophane disk (Hutchinson, Chalette-sur-Loing, France) was layered on a solid ISP2 medium before *A. flavus* inoculation. After the incubation, the mycelium was separated from the medium by removing the cellophane disk. Then, the cellophane disk with the mycelium was dried in an oven for 3 days at 60 °C before being weighed. For both methods used, *A. flavus* was inoculated as previously described on a solid ISP2 medium supplemented with CFS4 at the final concentration of 1.5 g/L and incubated for 5 days at 28 °C. As a control condition (Ctrl), *A. flavus* was inoculated on a not-supplemented solid ISP2 medium. The experiment was conducted twice in triplicate.

#### 5.6.2. Effect on *A. flavus* Sporulation

To evaluate the effect of CFS4 on *A. flavus* sporulation, *A. flavus* was inoculated as previously described on a solid ISP2 medium supplemented with CFS4 at the final concentration of 1.5 g/L and incubated for 5 days at 28 °C. At the end of incubation time, the total number of fungal spores from the entire fungal colony was counted according to the method previously reported by Caceres et al. [23]. Spore quantities from *A. flavus* inoculated on not-supplemented solid ISP2 medium (Ctrl) and supplemented solid ISP2 medium with the CFS4 at 1.5 g/L were compared. The experiment was conducted twice in triplicate.

### 5.7. Determination of the Half-Inhibitory Concentration (IC50) of CFS

The IC50 was defined as the concentration causing a 50% decrease in AFB1 or AFB2 concentrations in comparison with the control condition (i.e., *A. flavus* inoculated on not-supplemented ISP2 medium). For this, the following CFS4 concentrations were tested: 0.0005, 0.005, 0.05, 0.1, 0.187, 0.375, 0.75, 1.5, and 3 g/L. The volume of CFS4 added into the ISP2 medium was determined on the basis of its dry matter content. Then, *A. flavus* was inoculated as previously described and incubated for 7 days at 28 °C. At the end of the incubation period, AFB1 and AFB2 in *A. flavus* cultures were extracted, as described below. The experiment was conducted twice in triplicate.

### 5.8. AFB1 and AFB2 Extraction and Quantification by UHPLC/DAD/FLD

AFB1 and AFB2 from *A. flavus* culture were extracted from the entire medium (20 mL) and from the fungal colony according to the method previously reported by Caceres et al. [23]. The detection and quantification of AFB1 and AFB2 were performed using a Dionex Ultimate 3000 UHPLC system coupled with a diode-array detector (DAD) and a fluorescence detector (FLD) (Thermo Fisher Scientific, Illkirch, France). AFB1 and AFB2 analyses were performed using a Luna C18 column (3 µm, 150 × 4.6 mm) (Phenomenex, Torrance, CA, USA). A 20 min isocratic mode was delivered at 82.5% of eluent A (0.2% acetic acid/water and acetonitrile: 79:21; *v*/*v*) and 17.5% of eluent B (pure methanol). AFB1 and AFB2 were detected using FLD with 365/440 nm excitation/emission wavelengths and confirmed by their retention time (min) according to a standard (Merck KGaA), and peak identity was confirmed by analyzing absorption spectrum with a DAD coupled with the system. AFB1 was quantified by measuring the peak area according to a standard curve, with concentrations ranging between 20 and 300 µg/L. The limits of quantification were 5 and 15 µg/L for AFB1 and AFB2, respectively.

### 5.9. Microarray Gene Expression Studies

The fungal strain *A. flavus* was cultured on an ISP2 medium and incubated for 7 days at 28 °C in the dark. Then, a spore suspension was made and calibrated to 10^6^ spores/mL. Petri dishes containing ISP2 supplemented with CFS4 at the final concentration of 1.5 g/L were covered with sterile cellophane sheets and inoculated with 10 µL of the spore suspension. As a control condition, Petri dishes containing ISP2 not-supplemented (Ctrl) were also inoculated in the same way. Then, fungal cultures were incubated at 28 °C for 5 days. At the end of the incubation time, the entire fungal colony obtained was separated from the medium and ground up under liquid nitrogen. The RNA extraction and quantification were achieved according to the method previously reported by Caceres et al. [23]. RNA concentrations were quantified using a Nanodrop 2000 spectrophotometer (Thermo Scientific, Illkirch, France), and RNA integrity and purity were checked using the Experion RNA analysis kit (BioRad, Marnes-la-Coquette, France) and software (version 3.20, 2015, BioRad).

Gene expression profiles were performed at the GeT-tRiX facility (GénoToul, Génopole Toulouse Midi-Pyrénées) using Agilent Sureprint G3 Custom GE microarrays (8 × 60 K, design 085497) following the manufacturer’s instructions. For each sample, Cyanine-3 (Cy3), labeled cRNA, was prepared from 200 ng of total RNA using the One-Color Quick Amp Labeling Kit (Agilent Technologies) according to the manufacturer’s instructions, followed by Agencourt RNAClean XP (Agencourt Bioscience Corporation, Beverly, Massachusetts). Dye incorporation and cRNA yield were checked using Dropsense™ 96 UV/VIS droplet reader (Trinean, Belgium). An amount of 600 ng of Cy3-labelled cRNA was hybridized on the microarray slides following the manufacturer’s instructions. Immediately after washing, the slides were scanned on Agilent G2505C Microarray Scanner using Agilent Scan Control A.8.5.1 software, and the fluorescence signal was extracted using Agilent Feature Extraction software v10.10.1.1 with default parameters. Microarray data and experimental details are available in NCBI’s Gene Expression Omnibus [87] and are accessible through GEO Series accession number GSE232607 (https://www.ncbi.nlm.nih.gov/geo/query/acc.cgi?acc=GSE232607).

### 5.10. Analysis of Specialized Metabolites by LC-HRMS

Extracts obtained from the entire medium and fungal colony were resuspended in 500 μL of an acetonitrile/water mixture (50:50, *v*/*v*), actively shaken for 30 s, and thereafter sonicated (Bransonic 221 Ultrasonic bath, Roucaire, Les Ulis, France) for 2 h. A volume of 250 µL of 100% acetonitrile was added to each extract, followed by active shaking for 30 s and centrifugation (a few seconds). Specialized metabolite analysis was carried out using Acquity HPLC (Waters, Saint-Quentin-en-Yvelines, France) combined with an LTQ Orbitrap XL high-resolution mass spectrometer (Thermo Fisher Scientific, Les Ulis, France). Ten μL of the supernatant was injected into a 150 mm × 2.0 mm C18 Luna^®^ 5 μm reversed-phase column (Phenomenex, Torrance, CA, USA). Phase A was water acidified with 0.1% formic acid, and phase B was 100% acetonitrile. The following elution gradient was used: 0 min 20% B, 30 min 50% B, 35 to 45 min 90% B, and 50 to 60 min 20% B at a flow rate of 0.2 mL/min at 30 °C.

Acquisitions of HRMS were performed with electrospray ionization (ESI) in both positive and negative modes, as previously reported [88]. MS/MS spectra were obtained with collision-induced dissociation mode at low resolution and a normalized collision energy of 35%.

The metabolites were identified based on their MS/MS spectrum, MS/MS fragmentation pattern, and retention times, compared with those of the standard compounds or data from AntiBase 2012 or the literature [69,89].

### 5.11. Statistical Analysis

First, the normal distribution of data was tested by the Shapiro–Wilk test. Then, in experiments where the effects of the three CFSs (CFS4, CFS5 and CFS6) were compared with the control condition, one-way ANOVA, followed by Dunett’s multiple comparisons post-hoc test, was used. In experiments where the effect of CFS4 exposition was compared with the control condition, the Student’s *t*-tests followed by a Fischer test on the equality of variance were used. Differences were considered to be statistically significant when the *p*-value was lower than 0.05. Graphical values are represented by mean ± standard error of the mean (SEM). The statistical analysis of data was carried out with GraphPad Prism 8 software (GraphPad 169 Software, La Jolla, USA).

## Data Availability

Not applicable.

## Supplementary Materials

The following supporting information can be downloaded at: https://www.mdpi.com/article/10.3390/toxins15070428/s1, Table S1: Details of gene expression regulation of the 56 secondary metabolites gene clusters of *A. flavus* in response to CFS4 exposition. Table S2: Relative abundances mean of specialized metabolites produced by *A. flavus* in control (Ctrl) and CFS4 condition, obtained by LC-HRMS.
