# Supplementary material for: Effect of Streptomyces roseolus Cell-Free Supernatants on the Fungal Development, Transcriptome, and Aflatoxin B1 Production of Aspergillus flavus
Source: Toxins (Basel). 2023 Jun 30;15(7):428. doi: 10.3390/toxins15070428 (PMC10467112; doi:10.3390/toxins15070428)
Supplement: Supplementary file 1 [file toxins-15-00428-s001.zip › Table S2 VF.pdf]

Table S2. Relative abundances means of specialized metabolites produced by *A. flavus* in control (Ctrl) and CFS4 condition, obtained by LC-HRMS ND: not detected

Specialized metabolite	Relative abundance means (± SD)		*p*-value
	Ctrl	CFS4	
Aflatoxin B1	5.21E+08 (± 3.53E+07)	2.49E+08 (± 1.35E+07)	<0.001
Cyclopiazonic acid	7.69E+08 (± 7.27E+07)	8.28E+08 (± 8.03E+07)	0.3197
Aspergillic acid	1.88E+06 (± 6.07E+05)	1.67E+07 (± 3.75E+06)	<0.001
Aspirochlorin	7.82E+06 (± 7.84E+06)	5.13E+06 (± 5.61E+05)	0.0007
Ustiloxin B	ND	ND	
